# A Good Death

**DOI:** 10.2196/jmir.9.1.e6

**Published:** 2007-03-14

**Authors:** David H Gustafson

**Affiliations:** ^1^Center for Health Enhancement Systems StudiesMadisonWIUSA

**Keywords:** Death, Alzheimer’s Disease, Hospice, Technology, End-of-Life Care, Intensive Care

## Abstract

The Institute of Medicine defines a good death a “one that is free from avoidable death and suffering for patients, families and caregivers in general accordance with the patients’ and families’ wishes.”. The current system creates barriers to reducing the stress and suffering that accompany a patient’s end of life. Data and eHealth technology, if it were more accessible, could help patients, families, and caregivers to cope with end of life issues.

## Introduction

Mom had Alzheimer’s. She was barely hanging on to her apartment in an assisted living facility and would soon have to move to an Alzheimer’s unit. Then breathing troubles started. In three days she was diagnosed with pneumonia, developed congestive heart failure, had a heart attack (one day after being admitted to the hospital), and died. In those three days more money was spent “caring for her” than had been during her entire life.

My dad died when I (the oldest) was 11. Mom raised three kids on a secretary’s salary, put three kids through college, and did so with jokes, smiles, and songs. Her view of life was: make lemonade out of lemons; get on with it; don’t complain.

She took the same approach to death. She got sick on Saturday, spent Sunday and Monday in an Intensive Care Unit, and died Tuesday noon. Even 30 minutes before she died she was trying to make it easy on us by joking. Then she waited to die until we went for to lunch because (I think) she did not want us to see her die. Like always, she did her job and moved on. She was 87. I loved her very much.

## A good death?

The Institute of Medicine defined a good death a “one that is free from avoidable suffering for patients, families and caregivers in general accordance with the patients’ and families’ wishes” [1]. I wish I could say my mother got the care she needed and deserved. She did not; neither did we. Those whom I told our experiences suggest they are all-too-common. Hence, I would like to share these experiences here, and discuss the needs, but also responsibilities, of patients and family members at end of a life, and the implications for the health field.

She was lying in that bed. When she first arrived at the hospital, she was still able to walk. I know that even at my age, I can’t go as long without physical exercise as I used to. When I take time off and get back to it, my muscles are sore. How long could she stay in that bed before she would no longer have the muscles to walk out? Would she not be able to return to her assisted living apartment? What about her heart attack? How much would that limit her?

Even in her debilitated mental state, she was probably the most rational person of us all. Mom knew what she wanted: to have the restraints and tubes *removed* and to go home. We were told that we could have none of that. The pneumonia had to be treated. The restraints had to stay on. The tubes had to stay in. Could her antibiotics be given by intramuscular or by pill? What if *we* took out the tubes, stood at the door and told the nurses to leave our mother alone? What would they have done? What rights do family members have and how could we exercise them?

The thing that bothered me the most was those restraints. I wonder if my mother would have had the heart attack if she had not been placed in restraints. Did the hospital “kill” her? Did the heart attack kill her? Or did she die of a broken heart? Let me explain. I was told that when Mom got to the hospital, she kept pleading to go home. Over and over and over again. The Alzheimer’s had made it difficult for her to deal with change and when the nurses added the IVs, she became combative. A team of nurses had to use restraints to subdue a frail 87-year-old woman! Straps held down her hands so she would not pull out the IVs. Mom hated lying on her back, but the restraints made it impossible for her to lie on her side or even to scratch her nose. She continued to fight the restraints for several hours, all the time asking why she was in the hospital and why she could not go home. After a while, she was just exhausted, and shortly after that, she had a heart attack. If I were 87, begging to go home, and doing everything I could do to fight restraints, how long would my heart hold out? Did this have to happen? What rights did the family have? How could we have intervened? Looking back, I wonder what would happen if everyone working in an ICU were required to lie in restraints for just one hour.

I found that not all “do not resuscitate” (DNR) orders are the same. A nurse in the ICU told us that Mom’s orders were signed so long ago (seven years ago) that they were not valid. But now she had Alzheimer’s and, because of her limited cognitive functioning, had given up power of attorney for health care. Was the nurse right? How were we supposed to know that? What can we do now?

The ICU nurses said that my mother’s assisted living facility could not handle a person as sick as she. Again, we had so many unanswered questions. Is it illegal for an assisted living facility to provide such care, even on a temporary basis? Could our family have hired 24-hour nursing care and kept her in the assisted living facility? Where could we find good caregivers even if we were allowed to? How would we know a good home care nurse from a bad one? How would we monitor the care to ensure it was of high quality? What could we have done if poor care was provided?

The nurses told us we would have to raise our questions with the doctors. Yet they seemed to be avoiding us. We called the lead physician’s office many times and received no response. We even arrived very early (before 7:00 a.m.) to catch that physician on his rounds; he had “just left.” The few communications we did have with doctors were inconsistent and conflicting. The pulmonologist said Mom had 24–48 hours left. The nurses said she was looking better. An internist said she would be home in two days and a psychiatrist just appeared regularly to yell (literally): “Olive, do you know where you are? Can you spell your name?” I interrogated the cardiology nurse. She would not give a prognosis, so I asked “Was there tissue damage?” She said “Yes.” I said “Was it a minor heart attack?” She said “No.” I said “Was the damage was pretty significant?” She said “Yes.” I looked at Mom and thought that she did not look good at all. While she might have been more awake on the second day, she was fighting for air; her chest and abdomen going up and down. I could not see how she was getting better. How could I get a straight answer [2]? How could I take definitive action when I received conflicting advice and my own common sense told me that I was not getting a straight story? How could I get the doctors and nurses to talk to me? Who could I call if they don’t? Who was in charge?

It was difficult for us to know whether our mother’s condition was due to her disease or her treatment. Mom was clearly out of it. But she was on Haldol, which she had waited several hours to get while the staff attended to another emergency and while she was fighting her restraints. The nursing staff reported that the Haldol helped Mom quiet down. But when I first saw her, her speech was slurred and she looked awful. How could I know whether it was the Haldol or the heart attack or the lack of oxygen to the brain caused by pneumonia? No one would talk with us!

I kept asking myself: “What does Mom want?” Does she want to die? Mom was awake. I could have asked her. But how? Do I say: “Mom, do you want to die?” Or “Have you had enough?” I wanted to approach this the right way, but how do I open the discussion? AND, suppose she had said “Yes!” Then what? What could we have done to help her along?

Was it time to talk to hospice? The community offered several hospice options. Which one should we talk to? How could we find the best one? Was she eligible to go? What would they do for her? If hospice was available, it might be another difficult transition for Mom. I just wanted to ease her misery.

## Training medical professionals on the dying process

The death and dying process, including the needs of family members, should have a significant place in the training of physicians and nurses. But when I asked the nurse in charge of the ICU whether the hospital had a palliative care program, she replied: “What is that?” After we complained several times that we could not reach the physician, a “case manager” appeared, asking: “What do you want?” But we did not know what we wanted! She gave a list with telephone numbers of nursing homes and hospices but would not identify the good ones. When we asked whether the good ones were full and all the bad ones empty, she avoided the question. Again, there were so many unanswered questions. How could we get Mom into a nursing home or hospice that would be nice to her? How would we know if they were giving good care? How could we intervene if they weren’t? Time was getting short. We needed answers.

I wish that I could be optimistic that things will be better. I am not. The health field has recommended an array of end-of-life policies and best practices [3-6]. The Institute of Medicine and major provider associations, have issued reports, studies, and calls for change. The Robert Wood Johnson Foundation invested millions of dollars to promote a good death. But little has changed. The health care field is full of studies showing that patients’ wishes are not respected [7], that communication in the ICU is sorely lacking [8]; that evidence-based practices are not implemented [9]; and that fundamental concepts of palliative care (and even decency) are absent in many health care organizations [10].

Providers (and consumers) need to be more comfortable with uncertainty and with death. Long ago, Larry Weed (developer of the problem oriented medical record and Problem Knowledge Couplers) warned that it was not practical to expect a doctor to store and effectively process the vast and rapidly growing base of medical knowledge. In many cases, health care providers just don’t know how bad things are or the best course of action for a patient. So it is reasonable for a doctor to express uncertainty about what to do or whether the situation is dire enough to call the family together. But many providers act as if death is a failure, when it is part of life [11]. All patients and families deserve honest and consistent information, even if the information is an expression of uncertainty.

Healthcare providers are good people. I have seen my sister, a critical care nurse, come home from her job at a leading teaching hospital so tired she can barely move. I have seen doctors so frustrated with the health system that they can barely see straight. Providers are trained to cure at all costs but the incentives are to ensure that they spend as little time a possible with individual patients. I have seen hospital administrators after another long night of worrying on how they will make budget. If I were in their shoes with the same pressures and incentives, I would probably do the same thing. It should be different but it won’t; not for a long time. The problem is the system, not the people.

In the final analysis it is up to us (the families) to take more responsibility for the dying person and for ourselves [12]. We are the ones who care the most about what happens. We are the ones that will make the time. We need to know what to look for and what to expect. We need to know how to care for a patient at the end of life. We need to know our rights and how to exercise them; our options and how to choose between them. We need to know how to assess quality and how to act on those assessments. And we need to understand that death is part of life and that uncertainty is part of the dying process [13].

Ideally, families should be prepared to deal with death and dying well before the event. But it doesn’t work that way. I have done health services research for over 30 years. I currently have two research grants on death and dying. I knew my mother was approaching the end of her life. I should have been prepared for her death. I wasn’t. I was powerless when it came to making things happen in the ICU. I knew the principles but not the specifics of how to interact with a dying patient, and I needed the specifics. Our family (those in the ICU and those far away) needed ready access to information on Mom’s status as well as easy-to-find, easy-to-apply, just-in-time training on death and dying; training that was accessible while we sat in the intensive care unit and included the specific signs to look for and specific words to use with patients and providers.

## Recommendations

I have three suggestions: 1) automate the processes for helping patients and families deal with dying and death, 2) when automation is out of the question, make it hard for ICUs to do the wrong thing, and 3) improve transparency of how healthcare deals with death and dying.

### Automate

Given the pressures that health care providers operate under, it is unrealistic to expect training and exhortation to change anything. Technologies are needed that equip patients and families to deal with death and dying. Things would have been so much better if I could have opened my smart phone and pulled up a list of the 20 things to watch for in the ICU: things like restraints, conflicting information, care contrary to patient wishes, goals for end of life, family-physician communication. If I could have selected one of those things on my smart phone and seen an overview of the issues, my rights, specific steps I could take. If I could clicked on a topic and received more detail through decision aids, scripts, assessments, and training on how to exercise my rights, all presented in way that I could apply on the spot. If I could have had immediate Web access to databases on ICU, hospice and nursing home quality and relevant literature. If I could have sent a text message to an expert in death and dying or seen video clips of an effective encounter with a patient, a family member, a nurse, doctor or administrator. If I could have asked a question of other families who had gone through something similar or read stories of their experiences. The reality is that I *could have*—the technology, the knowledge, and the data exist to deal with most of these issues.

My wife and I just completed our Health Care Power of Attorney documents and sat down with the kids to review them. I wish that I could have handed the kids a memory stick containing that information, reviewed the basic structure with them, and asked them to carry it with them, because some day (5, 10, 30 years from now—or maybe tomorrow) they will need it, and so will I. Research and development could make this kind of tool a reality. But we can’t stop with development. We need to have a system for dissemination. Partnerships with the legal profession could ensure that when a will is prepared or updated that the participants and their families be given access to these tools. Hospitals could make this technology available to families whenever a patient is admitted to intensive care.

Secondly, electronic medical records (EMRs) could improve end of life. Is it out of the question to give families access to the medical record during this difficult time? Could EMRs place the patient’s goals for end of life care in an easy to access location that would be hard for any provider to miss? Could EMR’s have reminder systems that would alert providers when it is time to encourage patients to update their end of life wishes? Whenever a patient is considered to have a potentially life-threatening condition, could the EMR require documentation from ICU clinicians indicating that they have read and accept the patient’s goals for care at end of life?

### Make it hard to do the wrong thing

Systems change can be difficult. However, there are principles that can increase the likelihood of success. One is to remove the status quo. For instance, one cannot use mechanical restraints if they are not available. Administration could remove the mechanical restraints from the ICU and lock them in a cabinet for use only with permission of senior leaders. Medicare could treat inappropriate care at the end of life in the same way they treat other medical errors. Medicare policy could just flat out prohibit the use of mechanical restraints without permission from a senior leader of the hospital. Then, immediate convening of a rapid response team could be required to determine steps needed to remove the restraints and make sure they are never needed again. These steps have already been taken by mental health hospitals; we could do the same for dying patients. Technology could help to ensure that these policies are implemented.

### Transparency

Public reporting on the quality of a death should be required. Organizations like Medicare and NCQA could collect and publicly report data on use of restraints and other measurable dimensions of quality in death and dying. But public reporting is just a start. Systems must be in place to ensure that people will actually act on this information. Given our reticence to address death and dying before it happens, it is unrealistic to expect families to study quality of death data until the time comes. Hence, it will be important to find ways to make these data and resources easily available, easily understandable and easy to act on in a just-in-time basis.

We (the patients and families) need to take responsibility for our own dying and death. It is the centerpiece for us being able to do that. Mom needed it, I needed it and so will you.
